# How Therapeutic Tapping Can Alter Neural Correlates of Emotional Prosody Processing in Anxiety

**DOI:** 10.3390/brainsci9080206

**Published:** 2019-08-19

**Authors:** Nicola König, Sarah Steber, Josef Seebacher, Quinten von Prittwitz, Harald R. Bliem, Sonja Rossi

**Affiliations:** 1 ICONE—Innsbruck Cognitive Neuroscience, Department for Hearing, Speech and Voice Disorders, Medical University of Innsbruck, 6020 Innsbruck, Austria; 2 Department of Psychology, University of Innsbruck, 6020 Innsbruck, Austria

**Keywords:** electroencephalography (EEG), event-related brain potentials (ERPs), emotional prosody, anxiety, therapy research, Emotional Freedom Technique (EFT), Tapping, progressive muscle relaxation (PMR)

## Abstract

Anxiety disorders are the most common psychological disorders worldwide resulting in a great demand of adequate and cost-effective treatment. New short-term interventions can be used as an effective adjunct or alternative to pharmaco- and psychotherapy. One of these approaches is therapeutic tapping. It combines somatic stimulation of acupressure points with elements from Cognitive Behavioral Therapy (CBT). Tapping reduces anxiety symptoms after only one session. Anxiety is associated with a deficient emotion regulation for threatening stimuli. These deficits are compensated e.g., by CBT. Whether Tapping can also elicit similar modulations and which dynamic neural correlates are affected was subject to this study. Anxiety patients were assessed listening to pseudowords with a different emotional prosody (happy, angry, fearful, and neutral) prior and after one Tapping session. The emotion-related component Late Positive Potential (LPP) was investigated via electroencephalography. Progressive Muscle Relaxation (PMR) served as control intervention. Results showed LPP reductions for negative stimuli after the interventions. Interestingly, PMR influenced fearful and Tapping altered angry prosody. While PMR generally reduced arousal for fearful prosody, Tapping specifically affected fear-eliciting, angry stimuli, and might thus be able to reduce anxiety symptoms. Findings highlight the efficacy of Tapping and its impact on neural correlates of emotion regulation.

## 1. Introduction

Anxiety disorders are the most common psychological disorders worldwide with a lifetime prevalence of 5–30% depending on the country [1] and often they are not treated in an adequate amount of time. Among the most frequent reasons for this are lack of accessibility of treatment, especially in rural areas, long waiting lists for licensed psychotherapists or financial issues. Untreated anxiety disorders can result in a worsening of anxiety symptoms and the development of additional comorbid disorders like depression [2] or self-medication attempts [3], which can lead to many harmful consequences in the long run and decreases the likelihood of remission [2].

However, several short-term interventions have been developed in the past decades, which have shown to reduce symptoms of anxiety in only few sessions [4,5,6] and could be an approach to address this problem. They can be used in the context of anxiety treatment as an alternative or adjunct to pharmaco- or psychotherapy. One of them is therapeutic Tapping, a rather new approach based on a method known as Emotional Freedom Technique (EFT). It was developed by Craig and Fowlie [7] as a simplified form of thought field therapy (TFT) [8] and combines traditional Eastern techniques with modern Western therapy methods; it comprises elements used in Cognitive Behavioral Therapy (CBT) and Exposure Therapy, as well as somatic stimulation using 12 acupressure points distributed over the head, hand, and torso [9]. EFT is a combination of common western psychotherapeutic methods and principles also found in Traditional Chinese Medicine, kinesiology, and Eye Movement Desensitization and Reprocessing (EMDR). EFT in particular uses stimulation of specific acupressure points by Tapping them, which is done by the patient himself. This is why EFT is often simply referred to as “Tapping”. After professional instructions, it can be used as a self-help technique. Even though Tapping is gaining more and more popularity among clinicians worldwide, its active components have not been fully clarified yet. Until this date, there have been only few neuroscientific studies investigating the underlying cognitive mechanisms of Tapping in anxiety.

### 1.1. Altered Emotional Processing as a Key Aspect of Anxiety Disorders

Among the most important aspects of anxiety disorders are deficits in the regulation and inhibition of negative emotions [10,11]. Emotional regulation requires different cognitive functions, such as emotional processing and cognitive control. They are strongly interwoven, yet distinct constructs, which all seem to be affected by anxiety [12,13]. Anxiety is mediated by attention, in the sense that a stronger allocation of attention to the threatening event increases emotional arousal. As a consequence, a vicious circle maintaining or even increasing anxiety is activated [14,15,16]. The deficits in emotional regulation seem to be compensated by psychotherapeutic treatment like CBT [17], which might be a key factor in successful therapeutic treatment of anxiety.

### 1.2. The Late Positive Potential (LPP) as an Electrophysiological Correlate of Emotion Regulation

A well-suited measure of implicit emotional regulation is the assessment of its neural correlates, as they are not influenced by ongoing decision processes, in contrast to subjective behavioral measures. Electrophysiological signals provide a non-invasive measure of the electrical signal elicited by synchronous firing of neuron assemblies from the scalp. The advantage of electroencephalography (EEG) is the possibility to assess fast dynamic changes in the range of tens of milliseconds. EEG allows several evaluation procedures. One established analysis method is the extraction of event-related brain potentials (ERPs) from the spontaneous EEG. ERPs provide a stimulus-locked neural response sensitive to different experimental manipulations which can be extracted from the ongoing EEG by means of averaging several trials belonging to the same experimental condition [18]. This averaging technique allows the reduction of EEG background noise and provides ERPs differing in polarity, latency, topography, and sensitivity to experimental manipulation [19]. Emotional processes especially can be excellently tracked by means of ERPs. In adults, the neural substrates of emotional processing are mostly investigated with designs using visual stimuli such as emotional faces [20] or acoustic stimuli such as emotional vocalizations or speech prosody [21]. When investigating acoustic stimuli, speech-embedded stimuli are processed at a later stage than vocalizations [21]. This might be because the linguistic information in emotional speech cues requires more volitional capacities like attention and other mental resources [20]. Most stimuli in this context are constructed on the sentential level [21,22,23], or include real words instead of pseudowords [24,25] or contain explicit tasks [26]. However, an advantage of using pseudowords (imaginary words) over real words or sentences is that they do not carry any semantic content, which could confound emotional processing. Therefore, in the present study we opted for investigating acoustically presented pseudowords spoken in different emotional prosodic styles.

Apart from facial affect and emotional lexicon (i.e., semantic content of a word referring to whether the word per se describes a positive, negative or neutral affect. For example, the real word “pleasant” would semantically carry a positive emotion already in the word meaning), affective prosody is one of the three main channels to express an emotional experience [27]. This makes it an indispensable aspect of real-life communication, as a huge amount of emotional information is transported acoustically via speech intonation and melody. When investigating neural correlates of the processing of these emotional stimuli a typical electrophysiological measure is the centro-parietal Late Positive Potential (LPP; [28]. It is a slow positive deflection, which has been found to be enhanced during the presentation of emotional stimuli [29,30]. In general, a larger LPP amplitude indicates increased emotional arousal [31,32], reflecting subjective and objective emotional arousal measures [29]. Even when investigating only neutral pictures, more arousing pictures result in a larger LPP than less arousing neutral pictures [33]. Furthermore, the LPP amplitude was found to be strongly modulated by the perceived emotional intensity/valence of a stimulus. Stimuli with both a negative and a positive valence result in a larger (more positive) LPP compared to neutral stimuli [29,34,35]. The LPP typically peaks around 400–600 ms post stimulus onset [36]. However, it can also last up to 1 s after stimulus offset [37]. In most studies the LPP is elicited by pictures from the International Affective Picture System (IAPS; [38]) next to threatening faces or emotional gestures [39,40]. The LPP can be evoked with different presentation times, ranging from 5–6 s down to only 120 ms [41]. LPP differences can be found in adults as well as children to identify impaired emotion processing mechanisms [42,43,44]. These differences could be reliably observed in diverse clinical samples (e.g., depression, anxiety, schizophrenia, Parkinson’s disease). For example, reduced LPP amplitudes were found in Parkinson’s patients, where emotional reactivity is affected. This effect was especially noticeable when viewing unpleasant pictures [28]. LPP amplitudes to negative stimuli such as angry faces also seem to be directly and positively associated with symptoms of anxiety and depression [20]. Concerning factors which affect the temporal dynamics of the LPP one study found differences depending on the emotion regulation strategy [45]. When comparing the two emotion regulation strategies distraction and reappraisal, distraction reduced the LPP earlier than reappraisal.

Concerning the neural basis of the LPP, a simultaneous measurement of EEG and fMRI suggested that the LPP is generated and modulated by a brain network of cortical as well as subcortical structures responsible for emotional processing [46]. Results showed that the activity of the involved structures depends on the LPP amplitude and is valence-specific. When presenting unpleasant pictures, the ventrolateral prefrontal cortex (VLPFC), insula and posterior cingulate cortex were observed as neural generators of the LPP response [46]. For pleasant pictures it was the occipitotemporal junction, medial prefrontal cortex (PFC), amygdala and precuneus. A connectivity analysis in a study using magnetoencephalography (MEG) stated a functional connectivity between bilateral occipitoparietal cortex and right PFC [47]. This bidirectional connection was shown to be stronger with more arousing emotional content. However, it has to be noted that all of these studies presented visual stimuli (emotional pictures).

### 1.3. Neural Effects of Therapeutic Interventions on Emotional Processing

According to a study by Klumpp, Fitzgerald and Phan [48] with patients diagnosed with Social Anxiety Disorder (SAD) therapeutic success could be predicted by enhanced pre-treatment activation to threatening faces in higher-order visual, as well as cognitive and emotion processing areas measured by functional magnetic resonance imaging (fMRI). Twelve weeks of CBT led to the various brain changes when viewing emotional faces, e.g., in a reduction in activity in insula, prefrontal, and extrastriate regions [48]. Another study investigating emotional processing in SAD found a comparable LPP between patients and healthy controls during the presentation of negative images when using reappraisal [49]. This highlights the usefulness of the LPP not only as an indicator of arousal for the investigation of emotional processing, but also as a promising measure of emotion regulation and as therefore a potential indicator of therapeutic success. Reappraisal as a cognitive strategy to reduce negative affect is a technique used in CBT and also in Tapping as part of the affirmative statements.

### 1.4. Effects of Tapping in Anxiety Patients

A recent meta-analysis by Clond [9] found an overall effect size (Cohen’s d) for EFT-Tapping in the treatment of anxiety disorders of 1.23 (95% confidence interval [CI], 0.82–1.64; *p* < 0.001), indicating that Tapping might be a promising therapeutic intervention for this clinical sample. Wells et al. [50] found significant treatment effects after only one session of EFT when investigating subjects with specific phobia (diagnosed by means of the Diagnostic and Statistical Manual of Mental Disorders fourth edition - DSM-IV). So far, the description of possible Tapping treatment effects has mostly been restricted to behavioral data [50,51,52]. There are only few studies investigating electrophysiological correlates of EFT or its precursor Thought Field Therapy (TFT). However, these studies focused on oscillatory activity in the EEG without any experimental manipulation specifically addressing the assessment of cognitive and emotional processes and can thus only assess overall electrophysiological changes not directly related to a specific emotional state. Moreover, the assumptions about possible mechanisms in action of Tapping are yet to be supported by more scientific studies.

### 1.5. Aims of the Study and Hypotheses

The aim of this study was to examine the potential influence of Tapping regarding the regulation of emotions as one of the key functions being modulated by effective therapeutic interventions. Thus, we assessed electrophysiological signals while anxiety patients listened to acoustically presented pseudowords in neutral, angry, fearful, and happy emotional prosody. Subsequently, a therapeutic intervention was performed, followed by a posttest presenting new pseudowords, which corresponded to the same emotions as those in the pretest. Instead of the waiting list/no-treatment option for the control group, the Tapping intervention was compared to an established short-term intervention, namely Progressive Muscle Relaxation by Jacobson (PMR; for more details see Section 2.3). Based on the literature mentioned above we formulated the following hypotheses for this study:

After Tapping intervention EEG amplitudes will show a reduced Late Positive Potential (LPP) compared to before treatment in centro-parietal areas, indicating reduced emotional arousal after the Tapping intervention. This effect will be especially distinct for negative emotional prosody (angry and/or fearful stimuli).

If an involvement of cognitive reappraisal affects the LPP [24] we expect larger neural changes induced by Tapping compared to PMR, as Tapping includes cognitive reappraisal whereas PMR does not.

Thus, the present project is the first study to investigate neural changes in the processing of emotional prosody as a result of Tapping, especially in the case of anxiety patients and elucidate its active components. Neuroscientific measurements were conducted with electroencephalography (EEG), which bears the potential to assess dynamic changes occurring at a very fast time scale in the range of milliseconds.

## 2. Materials and Methods

### 2.1. Participants

**Figure 1:**
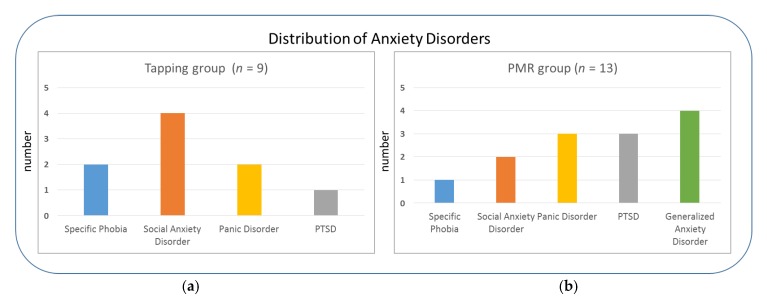
Main diagnoses of anxiety patients in the two intervention groups: (**a**) Tapping and (**b**) Progressive Muscle Relaxation (PMR); Post Traumatic Stress Disorder (PTSD).

Twenty-two German-speaking anxiety patients completed the study (15 female, 7 male). They had a mean age of 29 years (range: 19–52; *SD* = 8.4). All participants were recruited via public advertisement to ensure a representative sample of patients in terms of educational levels and socio-economic status. Inclusion criteria for the study were an anxiety disorder according the International Classification of Diseases 10th revision (ICD-10) diagnostic criteria [53] as main diagnosis (F.40, F.41 or F.43), based on the Mini-DIPS (Diagnostisches Kurz-Interview bei psychischen Störungen) interview ([54]; Figure 1). The severity of anxiety symptoms had to exceed a clinical cut-off of 4 on a scale from 1–8.

A mild to moderate depression (according to the Mini-DIPS interview and Beck Depression Inventory-II score, [55,56]) was the only comorbidity allowed, apart from additional anxiety symptoms or diagnoses. Participants with other psychological disorders, neurological disorders, hearing or visual impairments were excluded from the experiment. Written informed consent was obtained from all participants prior to measurements. In the present study patients received one of two anxiety treatments (Tapping or PMR), resulting in two treatment groups (Tapping: *n* = 9; PMR *n* = 13) after a pseudo-randomized assignment (see Section 2.4 for more details). Concerning age and gender both groups were balanced (mean age of 29 years in both groups; gender was compared with a chi-square test: χ^2^(1) = 1.12, *p* = 0.28, φ = 0.23). The two groups did not differ significantly (*p* > 0.05) concerning their levels of depressive symptoms (Beck Depression Inventory-II - BDI-II), current alertness (Karolinska Sleepiness Scale - KSS score) and anxiety (Numeric Analogue Scale - NAS score) before the pretest and posttest (see Table 1 for more details; for a detailed description on the used psychological tests please refer to Section 2.2.2).

Additionally, no differences in caffeine intake prior to the measurements or current medication were found between groups (see Table 2 for more details). NAS score (current anxiety level), KSS score (current alertness) and Positive and Negative Affect Schedule (PANAS, current emotional state) served as control variables before pre- and posttest to prevent effects due to sleepiness or just momentary states of more or less emotional agitation depending on the intervention. The total sample showed no overall differences between pre- and posttest in terms of sleepiness (KSS; *t*(20) = 1.759; *p* = 0.094) or emotional state (PANAS; *t*(21) = −0.650; *p* = 0.523). However, anxiety levels (NAS) dropped significantly in the total sample from pre- to posttest (*t*(20) = 3.833; *p* = 0.001). This reduction could be shown in both intervention groups when calculating *t*-tests within both groups (Tapping: *t*(7) = 2.646; *p* = 0.033; PMR: *t*(12) = 2.920; *p* = 0.013).

Participants received no compensation for participating in the study. The study was conducted in accordance of the Declaration of Helsinki and was approved by the local ethical committee of the Medical University of Innsbruck (Code: 1042/2017, 18 May 2017).

### 2.2. Materials

#### 2.2.1. Acoustic Stimuli

Sixty bisyllabic pseudowords (30 for pretest, 30 for posttest) were constructed. Onset consonant clusters conformed to the phonotactic rules of the German language and consisted of CCVCV (consonant-consonant-vowel-consonant-vowel) combinations. Five onset consonant clusters were chosen for pretest and posttest each: /br/,/**∫**n/,/**∫**t/,/kn/,/fr/(pretest) and /bl/,/**∫**w/,/**∫**m/,/kr/,/fl/(posttest). Six bisyllabic pseudowords were formed per onset cluster (e.g., braka, brefe, briti). They were constructed in a way, that each cluster included all five vowels and one alternating extra vowel ensuring the same distribution of vowels and consonants within pretest and posttest stimuli. The pseudowords were recorded in an anechoic chamber (Laboratory for Psychoacoustics at the Department of Hearing, Speech, and Voice Disorders of the Medical University of Innsbruck) by a female speaker. Each pseudoword was spoken and recorded in four different emotional prosodic styles: happy, angry, fearful, and neutral. The stimuli were recorded at 44 kHz and 16 bit sampling rate. Then they were edited using the editing program Audacity (www.audacityteam.org). This procedure included cutting, inserting a short silence period of 30 ms at each stimulus onset and offset, and normalizing pitch and loudness. Then they were acoustically presented in a pseudo-randomized order, according to the following randomization rules: maximally three pseudowords of the same emotional prosody in succession, in each experiment half each emotional prosody should occur equally often. Moreover, as different pre- and posttest stimuli were used to rule out any habituation or learning effects, they were tested for equal acoustic parameters. All pseudowords were analyzed by conducting ANOVAs with the factor emotion (neutral, angry, happy, fearful) and time (pre, post) on stimulus intensity, fundamental frequency and duration. The results of this analysis showed significant interactions between emotion and time for the parameters duration (*F*(3,87) = 9.012, *p* < 0.001) and fundamental frequency (*F*(3,87) = 3.104, *p* < 0.048). After Bonferroni correction post-hoc *t*-tests showed the following significant differences: only the duration for angry and happy prosody was longer for stimuli in the posttest compared to the pretest (angry: *t*(29) = −6.036, *p* < 0.001; happy: *t*(29) = −2.745, *p* = 0.010). No differences were present for fundamental frequency and intensity. Looking at the EEG curves, no latency differences could be attested between pre- and posttest (only differences in amplitude were present) indicating that a different duration of stimuli did not impact the electrophysiological signal.

**Figure 2:**
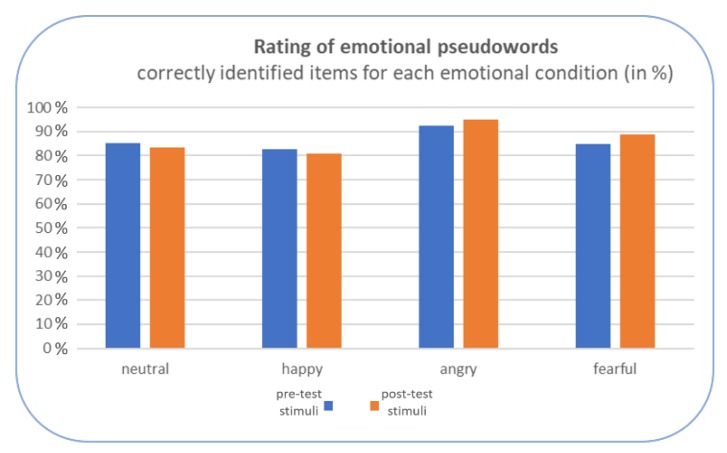
Results of the online rating of the acoustic stimuli.

To ensure that the intended emotional prosodies were identifiable, an online rating was conducted with an independent subject sample not taking part in the neuroscientific study. After listening to each stimulus, subjects had to rate it in terms of its emotional quality based on a choice of basic emotions. The overall identification rate of the emotions (merged across pre- and posttest-stimuli) was found to be as follows: neutral stimuli (84% correctly identified), happy (82%), angry (94%), fearful (87%), with no significant differences between pre- and posttest pseudowords in any emotional condition (*ps* > 0.050), see Figure 2.

#### 2.2.2. Standardized Psychological Tests and Additional Questionnaires

In addition to the neuroscientific measurements, all participants completed a number of standardized psychological tests and extra scales. The anxiety disorders were diagnosed by the Mini-DIPS interview [54] which was conducted with all participants in the study by specifically trained collaborators [57]. The Mini-DIPS is a German short structured clinical interview for diagnosing the most common mental disorders requiring therapeutic intervention. It is a well-established diagnostic instrument in clinical practice and scientific studies [58,59,60]. As a short version of the DIPS (Diagnostisches Interview bei psychischen Störungen) it can be conducted in 30 min on average.

Furthermore, other questionnaires were used to assess anxiety (Numeric Analogue Scale (NAS, scale 0–10), depression (Beck Depression Inventory-II (BDI-II); [55,56]), patients’ state of alertness (Karolinska Sleepiness Scale (KSS, [61]), and current emotional state (PANAS; [62]). The NAS is a simple Numeric Analogue Scale from 0–10 (0 = “no anxiety at all”, 10 = “highest anxiety level imaginable”) which was used over the course of the experiment to track changes in the perceived anxiety level. In the present study BDI-II, KSS, PANAS and NAS served mostly as control variables to check if the two intervention groups showed the same baseline characteristics in the light of a pseudo-randomized subject assignment. In addition, all participants completed a demographic questionnaire including the patients’ clinical history.

### 2.3. Therapeutic Interventions

The Tapping technique applied in this study is based on the Tapping method used in Emotional Freedom Technique (EFT) by Craig and Fowlie [7,63], which is an advancement and simplification of Thought Field Therapy (TFT), a former Tapping method by Callahan [8]. Thus, the correct term for the therapeutic intervention used in this study would be EFT-based Tapping, which is only referred to as Tapping or therapeutic Tapping in this article. It is a combination of elements used in cognitive behavioral therapy (CBT) and exposure therapy, as well as somatic stimulation using 12 acupressure points distributed over the head, hand, and torso [9], which are tapped by the patient himself. Therefore, the method is often simply referred to as “Tapping”. While stimulating these acupressure points one after the other, the subject describes and visualizes a certain distressing event or memory, which will then be broken down in its corresponding aspects and each connected with a reframing self-acceptance statement. These statements are usually set by the EFT practitioner and repeated several times by the patient. In this way, the patient keeps his attention on the selected problem while repeating the paired self-acceptance statement, which helps him to reframe the selected problem. Approaching anxiety in this way might also serve to detect and change internalized and often subconscious statements as part of a negative belief system. One of the most promising aspects of Emotional Freedom Technique is that it can be used as an addition to clinician-guided therapy, but also as a self-help technique after proper instruction by a Tapping professional. According to Craig [63], pairing affirmative statements with a relevant conflict can eliminate an anxiety disorder permanently. The technique can normally be mastered in only few guided sessions [5], which makes it very cost-effective and time-efficient. It seems to be a suitable intervention especially for the treatment of anxiety disorders [6,50,64]. In the present study, Tapping sessions were performed by a certified Tapping practitioner and psychologist.

Tapping was compared to Progressive Muscle Relaxation (PMR) by Jacobson [65], which also makes use of body-related experiences, takes a similar amount of time to be learned and can be applied as a self-help technique at home just as Tapping. It is a well-established method for anxiety disorders [66,67,68].

### 2.4. Procedure

Subjects were assigned to one of two intervention groups (Tapping or PMR) in a pseudo-randomized way to ensure no previous experience with the applied method.

**Figure 3:**
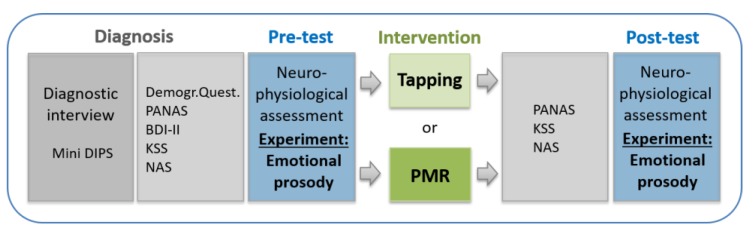
Experimental procedure including behavioral measures. Progressive Muscle Relaxation (PMR), Positive and Negative Affect Schedule (PANAS), Beck Depression Inventory-II (BDI-II), Karolinska Sleepiness Scale (KSS), Numeric Analogue Scale (NAS), Diagnostisches Kurz-Interview bei psychischen Störungen (Mini-DIPS).

Before measurements, each patient was diagnosed (or the currency of an existent diagnosis reevaluated) using the Mini-DIPS interview (see Section 2.2.2 for more details). Afterwards, participants completed several paper-based standardized psychological tests and additional questionnaires (for more details see Section 2.2.2 or Figure 3).

**Figure 4:**
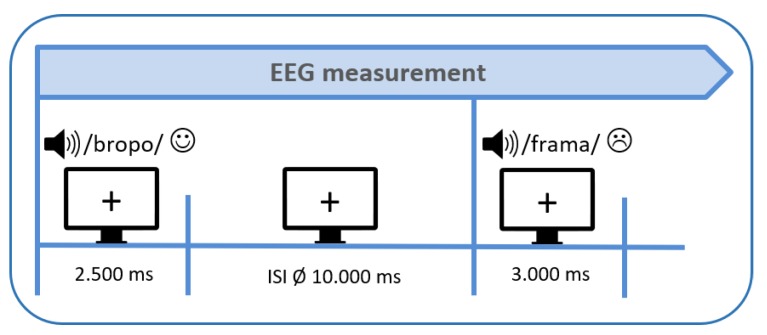
Experimental design (ISI = inter-stimulus-interval, EEG = electroencephalography).

Then the first measurement (pretest) with a simultaneous application of electroencephalography (EEG) and functional near-infrared spectroscopy (fNIRS) started. In the present paper we will, however, only report results obtained by means of EEG as there were some technical problems in the fNIRS data of some patients. Thus, we necessitate a larger sample size for reliable fNIRS data analyses. Participants were comfortably seated at an office workstation 1 m in front of a screen and instructed to listen passively to pseudowords spoken in different emotional prosodic styles. In total, 120 pseudowords were acoustically presented via stereo loudspeakers at an intensity level of 70 dB. Each trial started with the visual presentation of a fixation cross on the monitor for 2500 ms during which the acoustic presentation of the pseudoword started (see Figure 4). The fixation cross still remained on the monitor during the subsequent inter-stimulus-interval (ISI) which lasted 10 s on average (range 6–14 s). This extended ISI was introduced to minimize systematic overlap of the sluggish hemodynamic response measured by the fNIRS [69]. In total, the experiment lasted about 25 min.

After the pretest, one guided session of Tapping (experimental group) or Progressive Muscle Relaxation (PMR; control group) was conducted, lasting about 60 min each. After the intervention there was a standardized break of 15 min. Subsequently, participants of both groups completed PANAS, KSS, and NAS again to track potential changes over time. Then they performed the posttest, while undergoing the same experimental session as in the pretest. The whole procedure (shown in Figure 3) started between 10–11 am, which guaranteed comparable conditions for all subjects.

### 2.5. EEG Recordings

**Figure 5:**
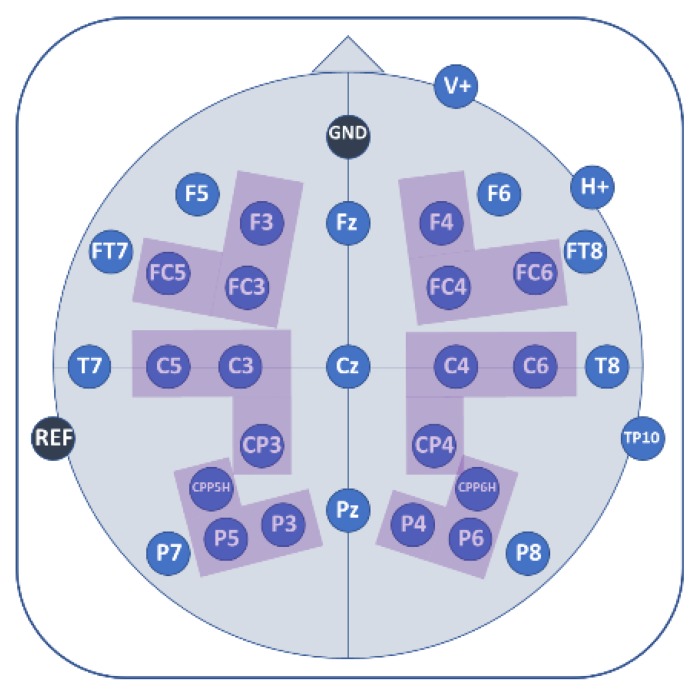
Electroencephalography (EEG) electrode placement. Colored areas indicate analyzed regions of interest (ROIs); consisting of three electrodes each.

EEG was recorded with 32 AgAgCl active electrodes (BrainProducts GmbH, Gilching, Germany). They were placed into an elastic EEG cap at the following positions: F5, F3, FT7, FC5, FC3, T7, C5, C3, CP3, CPP5H, P7, P5, P3, F4, F6, FC4, FC6, FT8, C4, C6, T8, CP4, CPP6H, P4, P6, P8, Fz, Pz, and Cz (Figure 5). Vertical and horizontal electrooculogram were recorded above and next to the right eye with electrodes FP2 and F10. One electrode (TP9) at the left mastoid served as online reference, while another one at the right mastoid (TP10) was recorded for further re-referencing during offline analyses. Position AFz served as ground electrode. Electrode impedance was kept below 10 kΩ (actiCAP Control, Brain Products GmbH, Gilching, Germany). The EEG signal was measured with BrainVision Recorder (Brain Products GmbH, Gilching, Germany) software with a sampling frequency of 1000 Hz (amplified between 0.016–450 Hz). It was filtered before digitalization by means of the analogue/digital converter with an upper cut-off of 450 Hz (24 db/oct) to prevent aliasing.

### 2.6. Data Analyses

#### EEG Data

EEG data was filtered offline with a 30 Hz low-pass Butterworth zero-phase filter (slope: 12 dB/oct). Then the data was segmented from −200 ms to 1500 ms with 0 ms representing the time of the pseudoword onset. After visual inspection of each segment for artifacts, overly contaminated channels were rejected manually and segment-wise. Only the data of those subjects in whom at least 2/3 of all segments per emotional condition in at least 25 out of 29 electrodes survived the artifact rejection process were included in the final analysis. The next steps consisted in re-referencing data to averaged mastoids (TP9, TP10) and applying a pre-stimulus baseline of 200 ms. Event related brain potentials (ERPs) were extracted by averaging the segments for each subject and each emotional condition.

**Figure 6:**
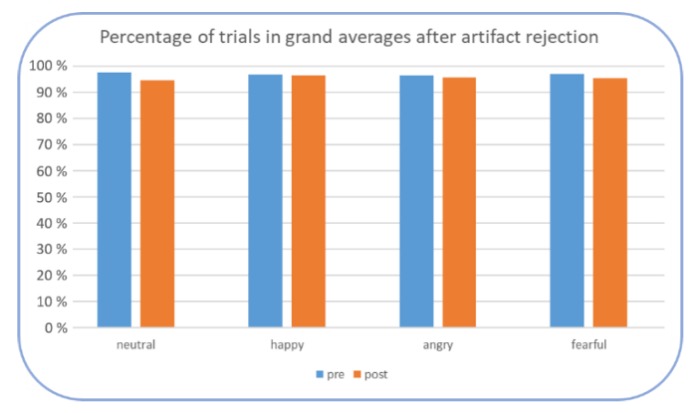
Mean percentage of included trials after artifact rejection in each emotional condition.

Furthermore, it was checked whether the amount of trials after artifact rejection was comparable across groups, emotions, and pre- and posttest. This was tested by means of an ANOVA with the within-subject factors emotion (angry, happy, fearful, neutral) and time (pre- and posttest) and the between-subject factor group (Tapping, PMR). No significant main effect or interaction were found (all *ps* > 0.050) indicating a similarly high percentage of trials in the final grand averages (above 94%), see Figure 6.

Subsequently, a 50-ms-analysis was performed in order to select the optimal time windows for final statistical analyses. This analysis included paired-sampled *t*-tests on each electrode between pre- and posttest per emotional condition in consecutive 50 ms steps between 100 and 1500 ms. After applying a Bonferroni correction for emotional prosodies, results from the 50-ms-analysis as well as visual inspection of the grand averages revealed five time windows: 150–250 ms, 250–350 ms, 350–650 ms, 650–1050 ms and 1050–1450 ms. Further statistical analyses were subsequently performed using the selected time windows.

Since the topographical localization of EEG is only rough, the final statistical EEG analyses were performed on 6 regions of interest (ROIs). These ROIs were defined as follows: left frontal (F3, FC3, FC5), right frontal (F4, FC4, FC6), left centro-parietal (C3, C5, CP3), right centro-parietal (C4, C6, CP4) left parietal (CPP5H, P3, P5), and right parietal (CPP6H, P4, P6) (Figure 5). Midline electrodes (Fz, Cz, Pz) were analyzed as single electrodes separately. The selection of ROIs was based on a common denominator relying on visual inspection of the grand averages and the highest correlation values obtained from correlation matrices of all electrodes over the averaged emotions in each time window included in the final analysis (see Appendix B, Figure A5).

Then a three-way ANOVA with the within-subject factors emotion (neutral, angry, happy and fearful) and time (pretest vs. posttest) and the between-subject factor group (Tapping vs. PMR) for all time windows was performed. Whenever an interaction with at least the two factors emotion and time reached significance, post-hoc *t*-tests were performed. Significance level was set at *p* ≤ 0.050. Corrected significance according to Greenhouse and Geisser [70] was applied whenever the degrees of freedom exceeded 1. Even though the mean age in both intervention groups was the same, considering the age range present in both groups it would have been interesting also analyzing a potential influence of age on neural processing, for example by means of mixed effects models. However, due to the rather small sample size in the two groups, this is not feasible.

## 3. Results

In the following, only those results where an interaction was significant that included at least emotion and time are mentioned.

For the time windows 150–250 ms, 250–350 ms, 350–650 ms, and 650–1050 ms, no significant results were obtained (all *ps* > 0.050).

**Figure 7:**
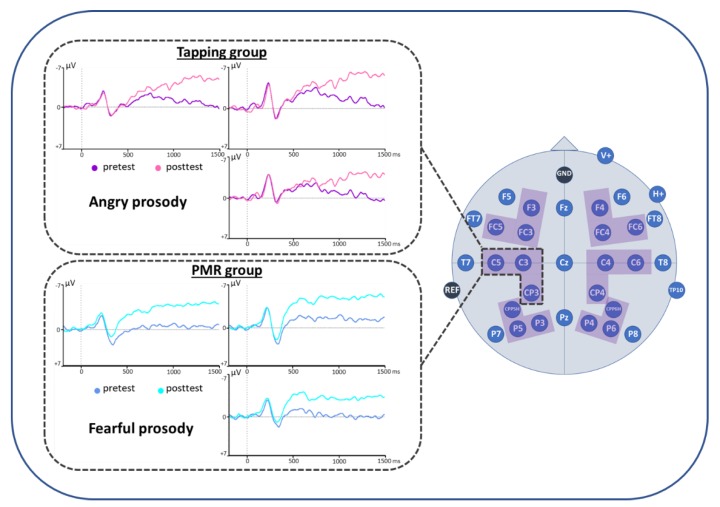
Event-related brain potentials (ERP) results. Positive polarity is plotted downwards.

**Figure A1:**
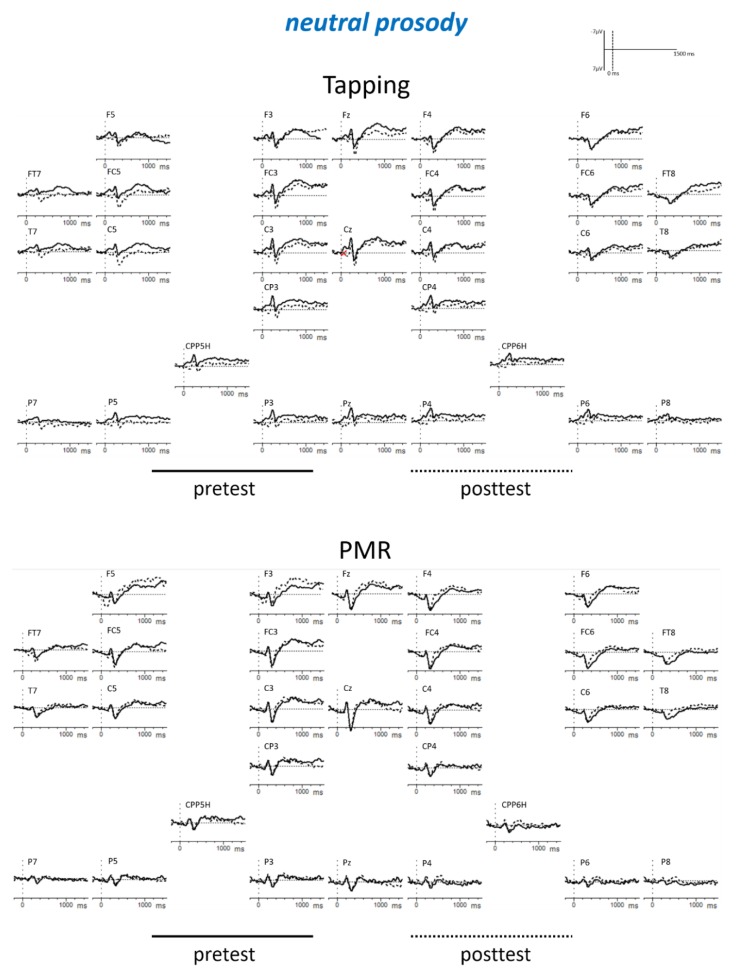
EEG results for all electrodes when processing neutral prosody. Positive polarity is plotted downwards.

**Figure A2:**
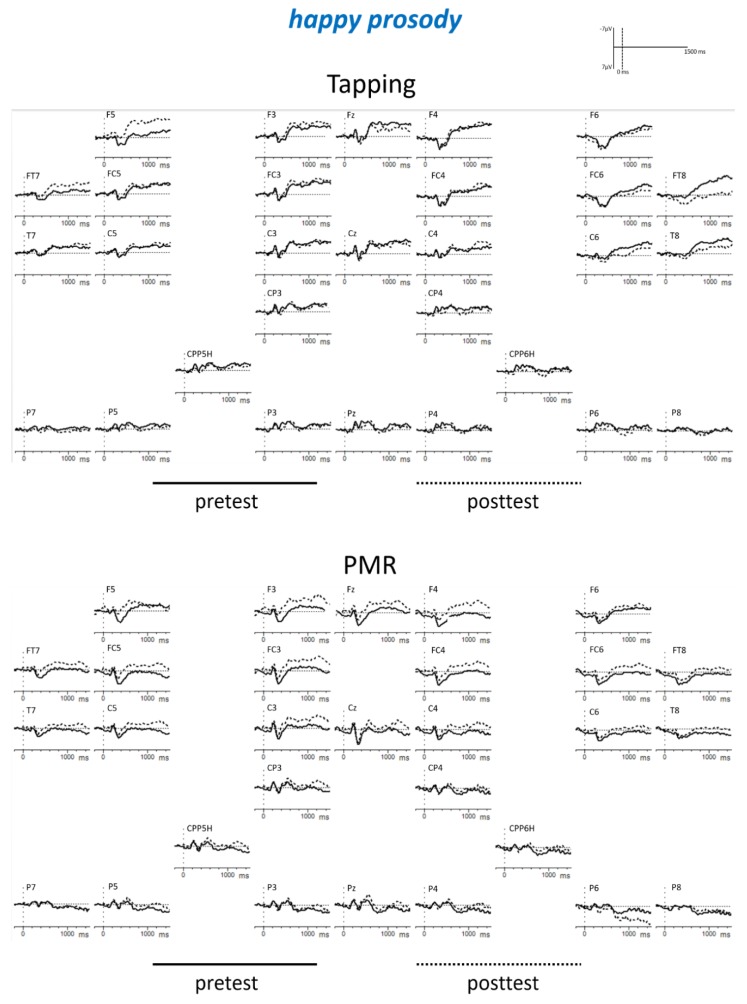
EEG results for all electrodes when processing happy prosody. Positive polarity is plotted downwards.

**Figure A3:**
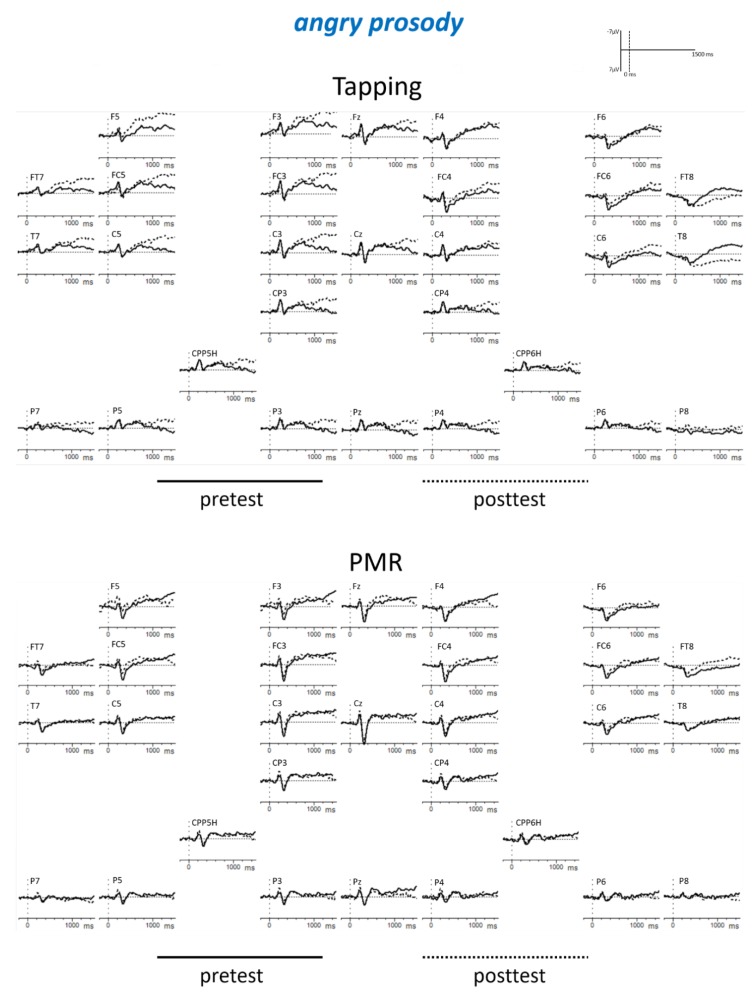
EEG results for all electrodes when processing angry prosody. Positive polarity is plotted downwards.

**Figure A4:**
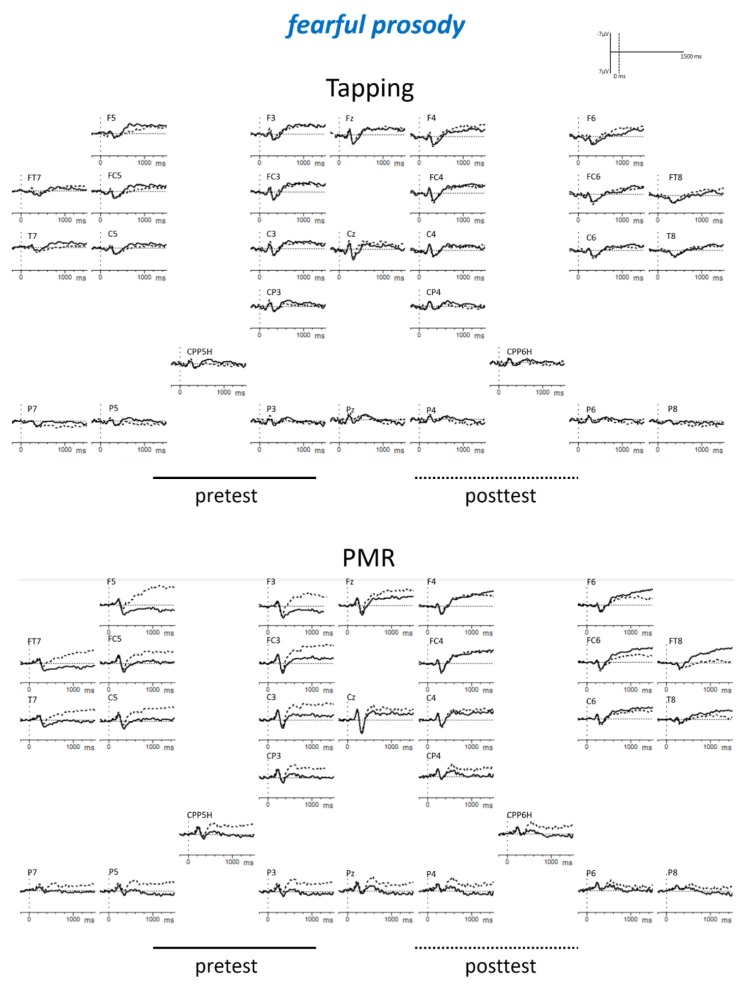
EEG results for all electrodes when processing fearful prosody. Positive polarity is plotted downwards.

**Figure A5:**
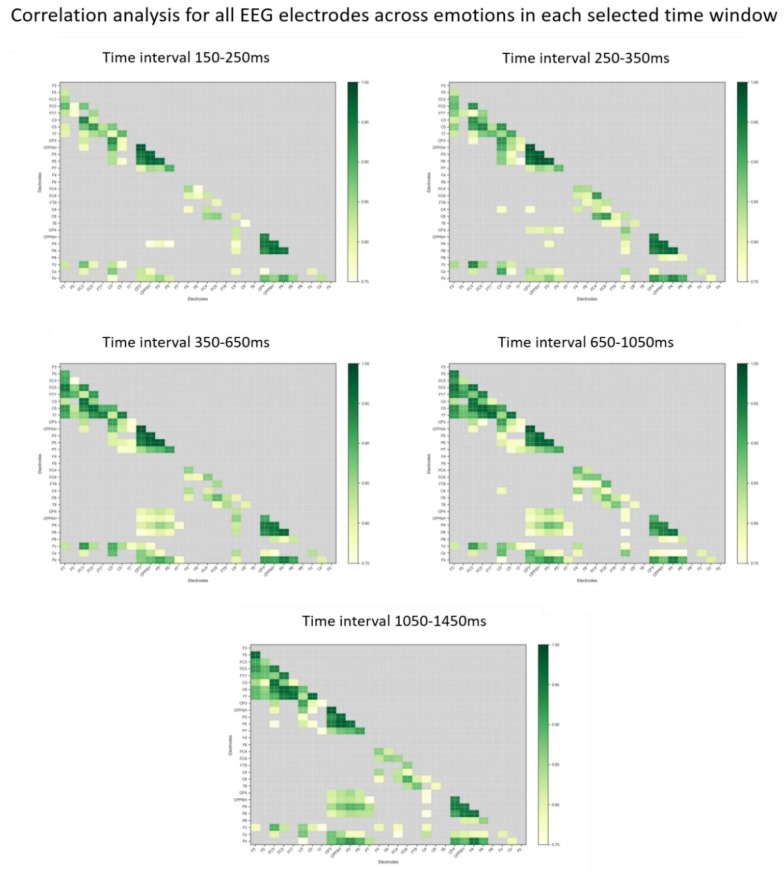
Selection of regions of interest (ROIs) based on correlation matrices of EEG electrodes for each selected time window at the baseline level (pretest). Pearson correlation with a clipping level *r*(avg) = 0.75.

For the time window 1050–1450 ms, the ANOVA showed a significant interaction of emotion x time x group on the left centro-parietal ROI (C3CP3C5) (see Figure 7 and Table 3). Post-hoc *t*-tests revealed a reduced positivity for posttest compared to pretest for the angry emotional condition in the Tapping group (*t*(8) = 2.003, *p* = 0.040). In the PMR group however, fearful stimuli led to a reduced positivity for posttest compared to pretest (*t*(12) = 1.953, *p* = 0.038). Accordingly, at the time of the posttest, fearful stimuli in the PMR group induced a smaller positivity than angry stimuli (*t*(12) = 1.806, *p* = 0.048). Also, compared to the Tapping group, the PMR group showed a reduced positivity for the processing of fearful stimuli at the time of the posttest (*t*(20) = 1.842, *p* = 0.040). All other ROIs in this time window did not reach significance. For an overview over the pre-post ERP responses for all groups and all emotions, please refer to Appendix A, Figure A1, Figure A2, Figure A3 and Figure A4.

## 4. Discussion

This study examined the neural effects of one session of therapeutic Tapping on emotional processing in anxiety patients. One session of Progressive Muscle Relaxation (PMR) served as control intervention. The results showed a reduced Late Positive Potential (LPP) in left centro-parietal areas while processing negative emotions after both interventions. The LPP is a typical ERP component found during the processing of emotional stimuli [29,31,32] with larger amplitudes for increased emotional arousal. Thus, both interventions seem to reduce arousal during the presentation of negative emotions. This might be an indication of improved emotion regulation, which is a key factor of successful psychotherapy [17,71]. Interestingly, the interventions affect different negative emotions as PMR mainly alters the processing of fearful stimuli while Tapping modulates the processing of angry stimuli. This finding might elucidate different mechanisms of action of PMR and Tapping intervention when treating anxiety.

In the present study, the LPP was mostly pronounced in relatively late stages of processing (the significant time window was 1050–1450 ms after stimulus onset). These later portions of the LPP might partly be attributed to an overlap of components that peak earlier and later across presentation time [72]. A similarly late LPP was also found in a study investigating neural reactivity to threatening faces in anxious youth [73]. The authors suggest that this finding might be due to sustained processing of threatening stimuli in anxiety, as anxious individuals show enhanced reactivity and sustained attention to threat compared to healthy controls. This goes in line with the fact that people with high trait anxiety generally show a heightened vigilance to emotional voices, which is not necessarily restricted to stimuli with an overt negative valence [13].

When interpreting the observed differences in the processing of negative emotions depending on the intervention type (Tapping or PMR) it is important to differentiate between the perception of an emotion, its expression or communication and the real phenomenological experience of an emotion ([74] for more details). Thus, emotional perception and the subjective emotional experience are two distinct processes, which can completely deviate from one another. While emotional perception refers to a more general way an emotional stimulus is processed, emotional experience describes the felt emotion elicited by a stimulus (usually named as a “feeling”), which is highly individual. For example, angry stimuli might elicit fear, whereas fearful stimuli might elicit pity. Empathetic responses to painful pictures were associated with larger LPP amplitudes [75], suggesting that a potential feeling of pity when presenting fearful stimuli could be observable in the LPP.

According to Gadea et al. [74], emotional experience comprises three components: valence (positive/negative or pleasant/unpleasant), arousal (from calming to arousing) and motor activation (ranging from approach to avoidance). Connecting these components with LPP amplitudes one study showed mainly arousal effects in the early epoch and valence effects in a later time epoch on LPP amplitudes [76]. This finding indicates that the late LPP differences for fearful and angry stimuli in the present study might predominantly reflect differences in the valence of these stimuli and therefore their emotional experience. This result seems plausible as a consequence of the distinct nature of the therapeutic treatments applied in this study; contrary to PMR, Tapping focuses on working intensely on the emotional level. Therefore, it facilitates and promotes the approach to negative emotions such as fear and anger. If we assume that the emotional experience of fear is rather elicited by angry than fearful stimuli, it is plausible that Tapping alters particularly the processing of these fear-eliciting, angry stimuli, as their reframing is an important part of the Tapping intervention. Consequently, the LPP might decrease. PMR however is a pure relaxation method promoting an overall state of relaxation in anxiety patients, which might generally reduce emotional arousal. Even though the earlier time windows did not result in significant LPP reductions for the PMR group, ERPs depicted in Figure 7 seem to suggest already an earlier initiation of the LPP reduction from about 400 ms onwards. This general arousal reduction might result in a feeling of pleasant indifference when confronted with fearful stimuli.

To further clarify these associations neural measures could be supported by additional behavioral data in future research. For example, by asking participants which emotion they perceive, and which emotion is elicited in them after each presented stimulus, including a measure of subjective valence and arousal to disentangle their assumed effects on emotional processing. However, it has to be considered, that the inclusion of such questions in the original experimental protocol could interfere with the measurement of truly implicit emotional processing mechanisms.

In the light of correlates between EEG results and clinical outcomes this study only provides first indicators, as the behavioral measures mainly served as control variables to ensure the comparability of patients and controls at the baseline level. Therefore, only the reduction in NAS scores from pre- to posttest could be interpreted as a result of the therapeutic interventions we applied. This reduction in the subjective current anxiety level from pretest to posttest (measured by means of a Numeric Analogue Scale, NAS) was found in the total study sample as well as in each group, irrespective of the applied intervention type. This is in line with electrophysiological results as the LPP reduces while processing negative emotions in both intervention groups. Therefore, the reduced anxiety score might be a first indicator for a behavioral therapeutic effect. However, it can only be interpreted as an instant change of an emotional state, even though other affective states did not change from pre- to posttest (no significant change in PANAS scores was found in both groups). Thus, further brain-behavior correlates of Tapping have to be clarified in a follow-up study. Then it will also be possible to track changes in BDI-II scores and more detailed instruments measuring anxiety over time to test the sustainability of potential therapeutic effects. A previous study with depressed patients (*n* = 17, out of which 10 were diagnosed with a comorbid anxiety disorder) showed a significant correlation of the change in BDI score with the change of the LPP after 15 months of long-term psychodynamic therapy [77]. It will be interesting if we can find such an effect as a result of Tapping or PMR as well at the time of a follow-up measurement.

Another aspect which should be considered in future studies is the inclusion of a control group without any intervention. This would further clarify the observed treatment effects, especially in terms of long-term effects and symptom changes by controlling for phenomena such as spontaneous remission. To avoid ethical concerns, a no-treatment control group could be realized by offering one session of Tapping/PMR after the end of the study. In general, the results of this study highlight the importance of distinguishing between specific negative emotional qualities such as fearful and angry prosody when investigating emotional processing in clinical samples. Moreover, it provides further evidence that auditory stimuli are equally suitable to investigate emotional mechanisms on a neural level. Investigating both channels of emotional expression (visual and auditory) is important for an extensive understanding of emotional processing mechanisms [27]. 

As Tapping induced neural changes in emotional processing in anxiety, it would be interesting to investigate its effects on other mental disorders as well, such as depression, schizophrenia or autism, which are also associated with an altered emotion regulation.

After all, this study provides first indications of the underlying neural mechanisms associated with altered emotional processing as a result of therapeutic Tapping sessions. Future investigations will have to additionally examine whether these changes only occur immediately and/or remain stable over time.

## 5. Conclusions

The present study compared two therapeutic interventions (Tapping and Progressive Muscle Relaxation - PMR) and their induced neural changes in anxiety patients. The Late Positive Potential (LPP) assessed by means of the electroencephalography revealed a reduction after the respective intervention. However, Tapping acted more on fear-eliciting angry stimuli while PMR predominantly modulated fearful stimuli. Thus, different emotional prosodies are tackled by the two interventions. This study further supported the notion, that the LPP component is a valid tool to investigate the effects of different therapeutic approaches.

## Figures and Tables

**Table 1:** Sample description of the two intervention groups at the time of the pretest (baseline).

	Tapping (*n* = 9)	PMR (*n* = 13)	*df*	*t*	*p*
Age (Mean)	28.56 (Range 20–52)	28.85 (Range 19–43)	20	−0.078	0.939
BDI-II score	13.0	13.23	18	−0.064	0.950
KSS score	4.25	4.69	19	−0.631	0.536
NAS	2.13	2.31	19	−0.325	0.748
PANAS	29.67	26.85	20	0.944	0.356

Beck Depression Inventory-II (BDI-II), Karolinska Sleepiness Scale (KSS), Numeric Analogue Scale (NAS), Positive and Negative Affect Schedule (PANAS), Progressive Muscle Relaxation (PMR).

**Table 2:** Sample description concerning clinical characteristics (based on Diagnostisches Kurz-Interview bei psychischen Störungen (Mini-DIPS) interview and demographic questionnaire).

Group	Additional Anxiety and/or Depressive Symptoms	Current Psychiatric Medication	Current or Past Psychotherapy
Tapping	78% ^1^	67%	67%
PMR (control)	85% ^2^	62%	85%

^1^ 45% of the Tapping group experienced additional anxiety symptoms to main diagnosis; 44% depressive symptoms, 11% somatoform symptoms. ^2^ PMR group: 69% additional anxiety symptoms to main diagnosis; 38% depressive symptoms, 8% somatoform symptoms, 8% subclinical eating disorder (related to anxiety).

**Table 3:** ERP results from the ANOVA emotion*time*group for the time window 1050–1450 ms at the left centro-parietal ROI (region of interest). Following significant interactions, the significant post-hoc *t*-tests are reported below. x < y indicates a reduced positivity for x compared to y.

1050–1450 ms Left Centro-Parietal ROI (C3CP3C5)	*df*	*t*	*p*
emotion*time	3.60	1.387	0.257
emotion*time*group	3.60	3.456	0.025
	Tapping: angry: post < pre PMR: fearful: post < pre PMR: posttest: fearful < angry Posttest: fearful: PMR < Tapping
